# The Future of Dialysis and Kidney Xenotransplantation: Promise, Cost, and Barriers to Implementation

**DOI:** 10.3390/jcm15218464

**Published:** 2026-10-30

**Authors:** Carmine Zoccali, Francesca Mallamaci, Giovanni Tripepi, Stefano Stuard

**Affiliations:** 1 Renal Research Institute, New York, NY 10065, USA; carmine.zoccali@icloud.com; 2 Istituto di Biologia e Genetica Molecolare (BIOGEM), 83031 Ariano Irpino, Italy; 3 Associazione Ipertensione Nefrologia e Trapianto Renale (IPNET), 89124 Reggio Calabria, Italy; francesca.mallamaci@libero.it; 4 National Research Council—Institute of Clinical Physiology, 89124 Reggio Calabria, Italy; giovanniluigi.tripepi@cnr.it; 5 Global Medical Office, Fresenius Medical Care, 26010 Palazzo Pignano, Italy

**Keywords:** end-stage kidney disease, hemodialysis, hemodiafiltration, medium cut-off membranes, peritoneal dialysis, precision dialysis, wearable artificial kidney, porcine kidney xenotransplantation, health economics and implementation barriers

## Abstract

**Background/Objectives**: Dialysis remains essential for patients with kidney failure but replaces only part of kidney function and imposes a substantial treatment burden. Gene-edited porcine kidneys offer a possible additional source of organs, although clinical experience remains limited. This review examines advances in dialysis and kidney xenotransplantation, their evidence base, costs, and barriers to clinical use. **Methods**: We undertook a targeted narrative update of clinical and technological reports, economic studies, and regulatory material. Randomized trials were considered separately from observational studies, target trial emulations, individual cases, preclinical experiments, and economic projections. **Results**: Randomized evidence supports a survival benefit of high-volume hemodiafiltration in suitable patients, although individual trials have differed and achieved convection volume is influenced by patient and treatment characteristics. Wearable dialysis and sorbent-assisted peritoneal dialysis remain at an early stage of clinical evaluation. Porcine kidney xenografts have provided dialysis independence in individual recipients, but rejection, endothelial injury, infection, and the burden of immunosuppression remain unresolved. Human kidney transplantation is generally favorable over longer horizons in eligible patients; its value depends on the setting. Xenotransplantation costs and long-term cost-effectiveness remain uncertain. Implementation requires financing, trained staff, reliable supply chains, and informed consent that addresses prolonged surveillance and cultural concerns. **Conclusions**: Improved dialysis and kidney xenotransplantation are likely to have complementary roles. Adoption should depend on demonstrated clinical benefit, sustainable costs, and equitable access, while prevention and early treatment of chronic kidney disease remain priorities.

## 1. The Changing Landscape of Kidney Replacement Therapy

Dialysis and kidney transplantation have carried end-stage kidney disease from inevitable lethality into a chronic, treatable disease. Neither, however, has resolved the fundamental limitations of kidney replacement.

Hemodialysis and peritoneal dialysis replace only selected excretory and regulatory functions of the kidney and remain expensive, infrastructure-intensive, and physiologically imperfect substitutes for kidney function.

Human kidney transplantation provides more complete physiological replacement and superior long-term outcomes, but access remains restricted by organ scarcity, comorbidity, age, sensitization, and major geographic inequalities.

**Figure 1:**
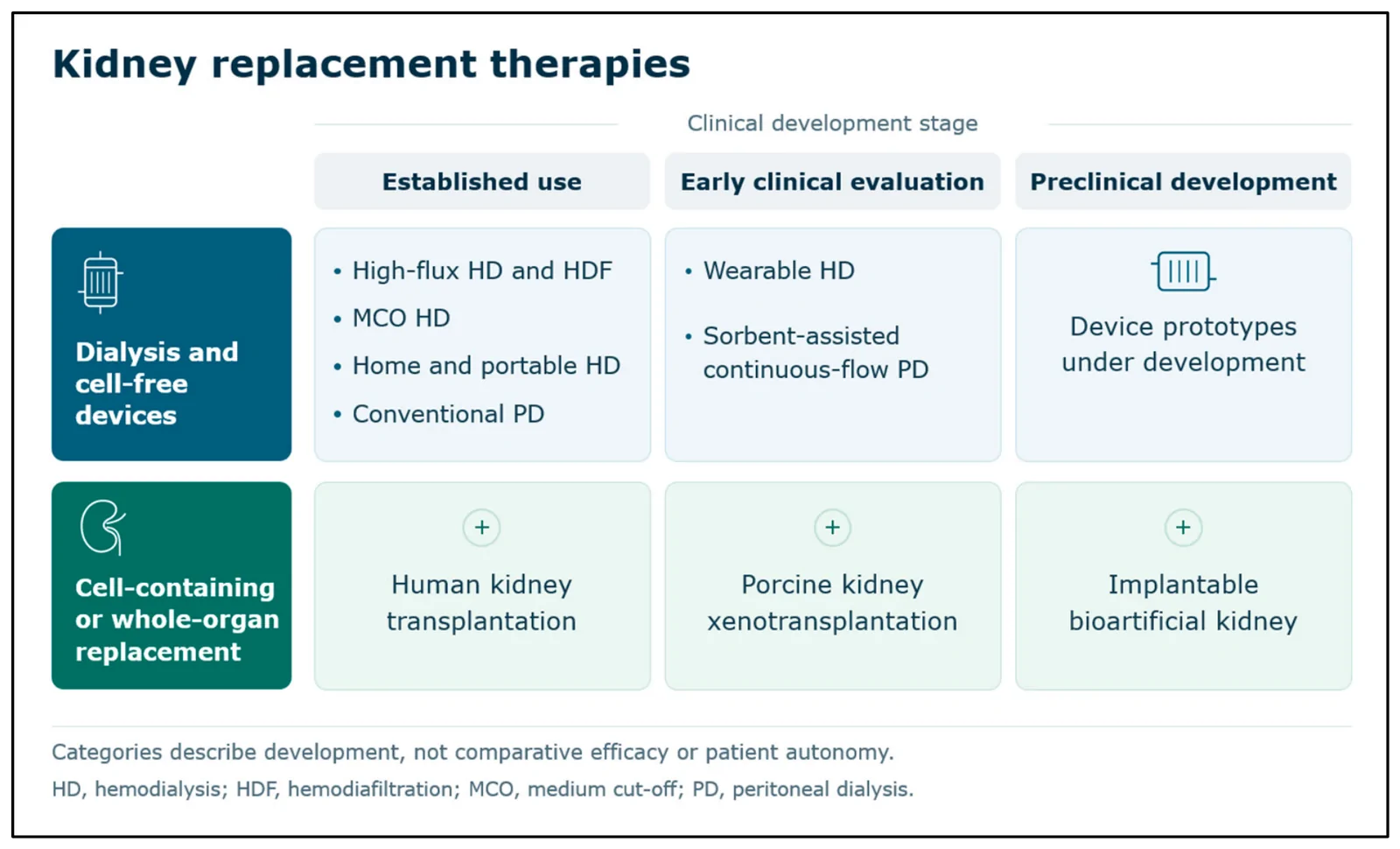
Kidney replacement modalities are grouped by clinical development stage and mode of replacement. The columns distinguish established clinical use, early clinical evaluation, and preclinical development. The rows distinguish dialysis and cell-free devices from cell-containing or whole-organ replacement. These are descriptive categories, not measured scales or rankings of efficacy, physiological function, autonomy, or quality of life. Established portable home hemodialysis systems are distinguished from experimental wearable systems.

Against this background, two technically sophisticated but conceptually different trajectories are emerging: The first seeks to improve dialysis by making treatment more physiological, individualized, continuous, and compatible with ordinary life, with further refinement and miniaturization of extracorporeal therapies. The second aims to expand transplantation by developing gene-edited porcine kidneys for clinical xenotransplantation (Figure 1). Both hold promise to reshape the field; both are constrained by formidable cost and implementation barriers. Table 1 summarizes the clinical evidence, proposed advantages, and main limitations of current and emerging kidney replacement modalities.

## 2. Literature Review

This narrative review was informed by searches of PubMed/MEDLINE [1] covering publications through 18 October 2026, without a lower date limit. Search terms included kidney failure, dialysis, hemodiafiltration, medium cut-off (MCO) dialysis, incremental hemodialysis, wearable artificial kidney, sorbent, continuous-flow peritoneal dialysis, and kidney xenotransplantation, combined as appropriate with randomized trial, immunosuppression, rejection, surveillance, cost–utility, cost-effectiveness, access, or workforce. The review prioritized randomized controlled trials, systematic reviews, individual-patient-data meta-analyses, regulatory documents, and studies reporting clinically relevant outcomes. Conference presentations were included when peer-reviewed reports were unavailable and were identified as preliminary; sponsor announcements were labelled as non-peer-reviewed sources. The review did not follow a registered systematic-review protocol and did not involve duplicate independent screening, formal risk-of-bias assessment, or a new meta-analysis. The search was not exhaustive, and the selection of evidence remains subject to selective retrieval and publication bias.

**Table 1:** Current and emerging kidney replacement modalities.

Modality	Evidence and Role	Function or Proposed Advantage	Main Limitations
High-flux HD	Established treatment [2]	Intermittent solute and fluid removal	Access complications, treatment symptoms, incomplete kidney replacement
HvHDF	Randomized trials, meta-analyses, clinical trial emulation, real-world evidence [3,4,5,6,7,8,9,10,11,12]	Diffusion plus convection; greater middle-molecule removal	Delivered convection volume depends on blood flow rate, treatment duration, and center expertise
MCO HD	Clinical use; preliminary randomized outcomes [13]	Larger middle-molecule removal through permeability and internal filtration	Albumin loss; uncertain long-term clinical benefit
Home HD, including portable systems	Established for selected patients [2]	Flexible schedules; reduced travel	Training, access safety, housing, supplies and support
Conventional PD	Established home therapy [14]	Solute and fluid removal without a blood circuit	Peritonitis, catheter or membrane failure, daily treatment burden
Wearable HD	Small, short feasibility trial [15]	Potential ambulatory, prolonged dialysis	Clotting, infection, sorbent exhaustion and device reliability
Sorbent-assisted continuous-flow PD	Early evaluation; CORDIAL protocol [16]	Sorbent regeneration; potentially lower dialysate use	Flow, infection, electrolyte control, cartridge capacity; uncertain long-term effects
Implantable bioartificial kidney	Preclinical development [17]	Filtration plus renal-cell activity	Cell viability, thrombosis, fouling, durability and replacement
Human kidney transplantation	Established for eligible patients	More complete kidney function; dialysis independence	Organ shortage, surgery, rejection, immunosuppression and graft failure
Porcine kidney xenotransplantation	Living-recipient reports; early trial programs [18,19,20,21]	Additional organ supply; potential dialysis independence	Rejection, endothelial injury, infection, immunosuppression, and prolonged surveillance; uncertain durability

HD, hemodialysis; HDF, hemodiafiltration; HvHDF, High-volume HDF; MCO, medium cut-off; PD, peritoneal dialysis. Development stages do not imply comparative efficacy. Reference [13] is a conference abstract; Ref. [16] is a trial protocol; Refs. [20,21] are sponsor announcements. Preclinical findings are distinct from outcomes in living recipients.

## 3. From Conventional Hemodialysis to Precision Dialysis

Current hemodialysis has achieved remarkable reliability and solute clearance, but remains predominantly center-based, intermittent, and disruptive to daily life. Rapid changes in fluid volume and solute concentrations may contribute to intradialytic hypotension, cardiovascular and cerebral stress, post-dialysis fatigue, and other treatment-related symptoms. A standardized prescription based primarily on small-solute clearance does not adequately account for differences among patients in residual kidney function, fluid status, vascular access, hemodynamic tolerance, nutritional status, comorbidity, frailty, and individual treatment goals.

### 3.1. Delivered Dose and Clinical Outcomes

Kt/V remains essential for assessing small-solute dialysis dose in hemodialysis with high-flux and MCO membranes, and hemodiafiltration (HDF), but does not capture middle-molecule burden or clinical outcomes.

Broader toxin removal should complement adequate small-solute clearance, and treatment assessment should also include measures of middle-molecule removal, treatment tolerance, symptoms, hospitalization, and long-term outcomes [2]. MCO membranes enhance middle-molecule removal through greater permeability and internal filtration and backfiltration, whereas HDF combines diffusion with controlled convection. Post-dilution HDF integrates diffusive and convective transport to enhance solute clearance across a broader molecular weight range than high-flux hemodialysis (HD).

Post-dilution high-volume HDF (HvHDF), delivering convection volumes >23 L per session, has been associated with lower all-cause mortality than high-flux HD [3,22,23]. CONVINCE provides randomized evidence supporting this approach, with a mean delivered convection volume of 25.3 L per session [3]. However, mortality findings have not been consistent across all HDF trials, which differed in delivered convection volume and HD comparator. Individual participant data meta-analyses of randomized trials support a survival benefit of HDF, with greater observed mortality reductions at higher convection volumes. A multinational target trial emulation involving nearly 20,000 patients, along with other observational studies, has reported similar associations [3,4,5,6,7,8,9,10,11,12]. The target trial emulation and other cohort studies provide observational evidence and remain susceptible to residual confounding. Furthermore, analyses of achieved convection volume are not randomized comparisons of convection dose. Delivered volume depends on treatment duration, blood flow rate, and center experience. Associations between achieved convection volume and survival may therefore partly reflect patient selection and treatment delivery. Preliminary results from the MOTheR trial did not establish non-inferiority of MCO dialysis versus HvHDF for all-cause mortality [13].

### 3.2. Volume Management and Residual Kidney Function

Precision dialysis requires regular assessment of hydration and hemodynamic tolerance, with individualized ultrafiltration, dialysate sodium and temperature, and treatment duration or frequency. Bioimpedance, blood-volume monitoring, automated ultrafiltration profiling, and other forms of biofeedback should complement clinical assessment.

Incremental dialysis may be appropriate for selected incident patients with substantial residual kidney function [24,25]. It requires timed urine collections to assess residual urea clearance, total weekly clearance, and urine volume, as well as monitoring of fluid status, blood pressure, potassium, bicarbonate, nutrition, and symptoms. Reassess residual function periodically and after acute illness or hospitalization. Declining clearance, recurrent hyperkalemia, persistent fluid overload, worsening acidosis, or uremic symptoms should prompt increased dialysis dose or frequency. Reduced treatment frequency without this monitoring is not an adequate incremental strategy. Precision does not necessarily mean greater intensity, but treatment must remain adequate as residual function declines.

### 3.3. Peritoneal Dialysis and Home Treatment

Peritoneal dialysis and home hemodialysis extend this principle by offering flexibility; in appropriate patients, more frequent or prolonged treatment may be preferred, but suitability depends on the patient’s clinical condition, home circumstances, training, and support. Peritoneal dialysis preserves residual kidney function and avoids the rapid extracorporeal volume shifts of intermittent hemodialysis, but its use is limited by peritonitis, membrane dysfunction, patient capability, and the transport and storage of large volumes of dialysate [14,26]. Peritoneal dialysis is also being revisited through technological innovation. Closed-loop or continuous-flow peritoneal systems that use sorbent cartridges for dialysate regeneration have been developed in experimental settings, enabling substantial reductions in dialysate volume and infrastructure requirements. The CORDIAL publication describes a first-in-human protocol for 12 stable adults, with six supervised treatments per participant; it does not establish long-term effectiveness [16]. Relevant uncertainties include catheter flow and recirculation, abdominal tolerance, intraperitoneal pressure, infection, cartridge capacity, electrolyte and glucose balance, and reliable fluid removal. Improved clearance during supervised sessions would not establish sustained benefit for peritonitis, membrane survival, hospitalization, or quality of life. Modality choice should continue to reflect clinical suitability and the patient’s informed preference.

Mathematical and computational models of peritoneal transport and lymphatic drainage are now being used to individualize dwell times and dialysate composition and to explore glucose-sparing strategies before clinical testing. Viewed together, these developments point toward dialysis treatments that are more continuous, mobile, and individualized. These systems are intended to reduce treatment burden; whether they improve cardiovascular tolerance, clinical outcomes, or patient-reported quality of life remains to be confirmed in appropriately designed clinical trials [16]. Modality choice should continue to reflect clinical suitability, home circumstances, caregiver support, employment, treatment burden, and the patient’s informed preference.

## 4. Portable, Wearable, and Implantable Systems

Within this landscape, the “artificial kidney” agenda has developed around two main lines: wearable and portable extracorporeal systems [15] and, at a more experimental stage, implantable bioartificial kidneys that combine synthetic filters with living renal cells [17].

Wearable hemodialysis systems use miniaturized pumps and sorbent-based dialysate regeneration to permit prolonged treatment with a closed, low-volume circuit [27,28].

Early human studies, limited by small samples and short duration, have demonstrated technical feasibility and short-term tolerability but identified vascular-access and device-related limitations that require further work. In the feasibility study by Gura et al., seven participants were enrolled and five completed the planned 24-h treatment; the trial was stopped after the seventh participant because of device-related technical problems, including excessive carbon dioxide accumulation in the dialysate circuit and variable blood and dialysate flows [15]. Potential benefits for hemodynamic stability and patient autonomy remain to be confirmed [15].

Miniaturization should not be confused with simplification. A wearable blood circuit must remain safe during movement and protect against clotting, disconnection, bleeding, and access infection. Sorbent capacity must be sufficient for the intended treatment period, with safeguards against exhaustion and, where urease-based regeneration is used, unwanted changes in dialysate composition. Sodium and volume balance require reliable measurements and control. Battery life, power failure, sensor drift, alarm performance, cartridge replacement, and maintenance all affect safety [27,28]. Patients and caregivers need training in troubleshooting and emergency disconnection, with a defined route to clinical support. Remote monitoring may assist supervision, but cannot replace local emergency arrangements or a safe device design.

If these obstacles can be overcome, more continuous treatment could reduce fluctuations in volume and solute concentrations and offer patients greater autonomy. The relevant outcome, however, is not simply whether a device can be worn. It is whether it can provide safe, effective, and sustainable treatment while reducing the intrusion of dialysis into the patient’s life.

## 5. Genetically Engineered Porcine Kidney Transplantation

Gene-edited porcine kidneys could increase the number of organs available for transplantation. However, avoidance of hyperacute rejection is only the first requirement. Sustained function also depends on control of cellular and humoral immunity, endothelial and coagulation injury, and infection [29].

### 5.1. Donor Engineering and Clinical Experience

Over the last decade, advances in genome editing, immunosuppressive strategies, pathogen control, and donor-pig breeding have moved porcine kidney xenotransplantation from preclinical investigation to early clinical evaluation [29]. Genetic modifications include inactivation of porcine genes involved in glycan xenoantigen synthesis, including GGTA1, CMAH, and B4GALNT2, to reduce expression of major glycan xenoantigens, together with the insertion of human complement-regulatory, coagulation-regulatory, and immune-modulating genes [18,29]. In parallel, CRISPR–Cas9 has been used to inactivate porcine endogenous retroviruses throughout the pig genome, reducing an important potential risk of cross-species viral transmission associated with xenotransplantation [30,31]. These advances culminated in March 2024 in the first transplantation of a porcine kidney from a donor pig carrying 69 genomic edits into a living human recipient at Massachusetts General Hospital [18]. The patient died approximately seven weeks after transplantation, and the treating institution reported no indication that the death was related to the xenograft. Nevertheless, the clinical course demonstrated that hyperacute rejection could be avoided and that the xenograft could sustain kidney function in a living recipient [18]. Subsequent clinical experience, including a New Hampshire man who lived for 271 days with a porcine kidney before returning to dialysis, indicates that medium-term xenograft function is achievable [19]. The recipient developed endothelial injury and thrombotic microangiopathy after immunosuppression was reduced during a bacterial infection. Graft failure led to nephrectomy; a human kidney transplant followed 82 days later [19].

### 5.2. Rejection, Endothelial Injury, and Immunosuppression

Removal of major glycan antigens reduces recognition by pre-existing antibodies but does not eliminate humoral immunity to remaining porcine epitopes. De novo anti-pig antibodies may activate complement and injure the microvascular endothelium. A 61-day decedent study documented antibody-mediated rejection with increased donor-specific IgG on day 33; rejection was reversed with plasma exchange, complement inhibition, and T-cell depletion [32]. This provides mechanistic evidence, but observations in a somatically supported decedent do not establish long-term safety in living recipients.

Endothelial activation, complement injury, and species differences in coagulation regulation can contribute to microvascular thrombosis. Human complement- and coagulation-regulatory transgenes are intended to reduce these processes, but do not ensure normal endothelial function [18,32]. T cells, macrophages, and natural killer cells may also cause injury. In the first living recipient, a biopsy on post-transplant day 8 confirmed T-cell-mediated rejection, which was successfully treated by intensifying immunosuppression. Following treatment, adaptive immune responses were suppressed, whereas innate immune activation persisted; antibody-mediated rejection was not detected [33]. Monitoring should therefore combine kidney function, proteinuria, antibody testing, and histology rather than relying on a single rejection marker. The reported regimen in the first living recipient included rabbit antithymocyte globulin, rituximab, corticosteroids, and complement inhibition with ravulizumab, along with anti-CD154 costimulation blockade using tegoprubart, tacrolimus, and mycophenolic acid [33]. The later 271-day report also used costimulation blockade with complement inhibition [19]. These remain experimental regimens. Their intensity requires assessment of opportunistic infection, cytopenias, metabolic toxicity, and the consequences of reducing treatment during infection. Protocols differ between donor platforms and recipients; no single regimen has established durable efficacy with an acceptable long-term toxicity profile.

### 5.3. Regulatory Evaluation and Infection Surveillance

The United Therapeutics EXPAND study (NCT06878560) and the eGenesis RESTORE program are described as phase 1/2/3 development programs, rather than simply phase 1 trials. The EXPAND sponsor report describes an initial six-recipient cohort, staged safety review, 24-week assessment, and lifelong follow-up. It reported the first trial transplant in October 2025 [20]. In September 2026, eGenesis described a planned 33-patient RESTORE study in selected adults aged 50–70 years, with initiation expected in 2027 [21]. The latter is a sponsor announcement about a planned trial, not a report of trial results. IND clearance permits investigation; it does not establish safety, efficacy, or approval for routine care. Small, selected cohorts and specialized-center care will limit generalizability.

**Figure 2:**
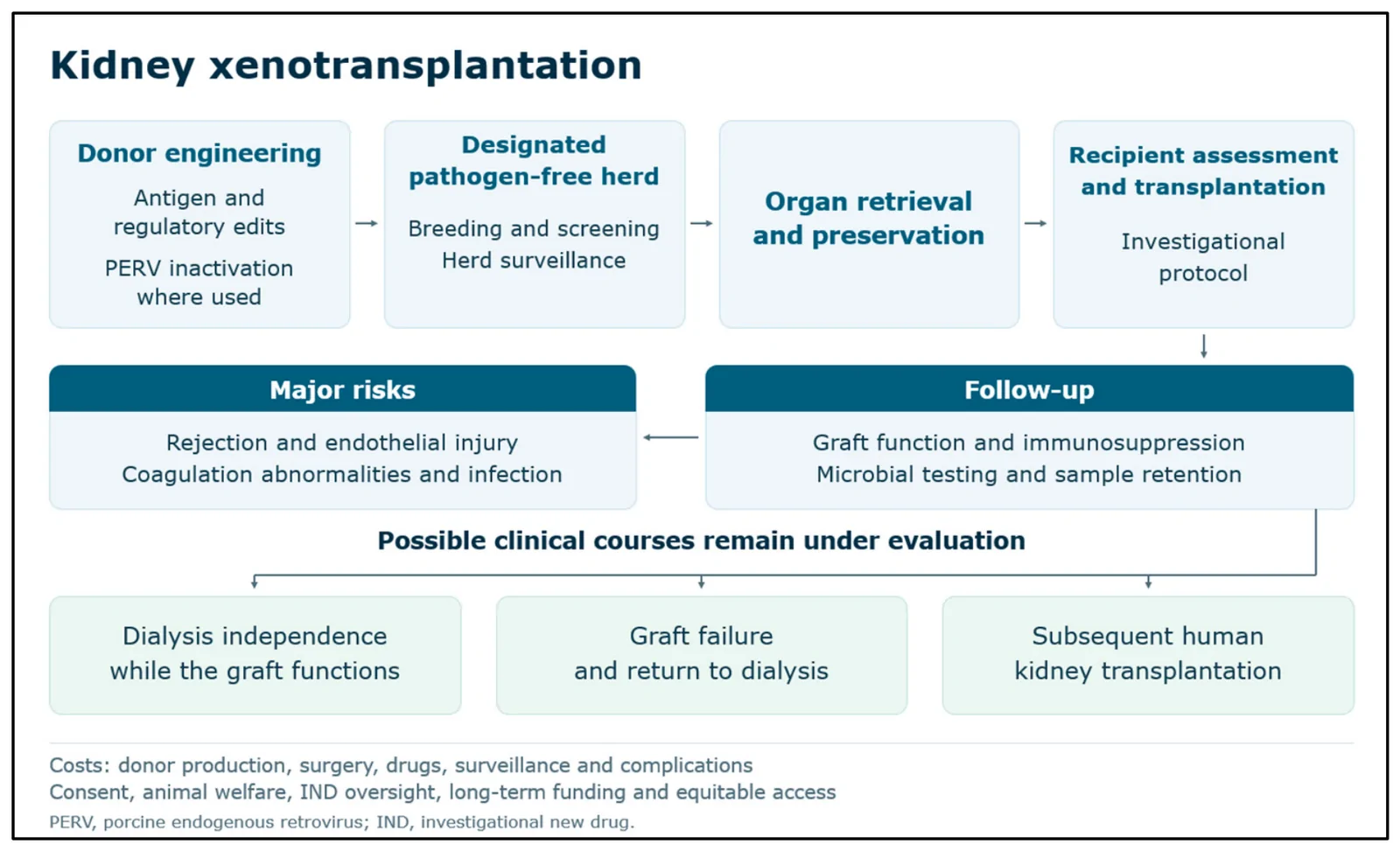
Clinical pathway for porcine kidney xenotransplantation. Donor engineering and designated pathogen-free breeding are followed by organ retrieval, recipient assessment, transplantation, and follow-up. PERV inactivation is platform-dependent. Rejection, endothelial and coagulation injury, and infection may interrupt graft function. Dialysis independence, return to dialysis after graft failure, and subsequent human kidney transplantation are distinct possible courses; their frequency and long-term consequences remain uncertain. Surveillance and sample archiving may continue after graft removal.

PERV inactivation addresses one potential source of infection; it does not eliminate other porcine pathogens or the possibility of previously unrecognized infection. FDA and US Public Health Service guidance recommend long-term or lifelong recipient surveillance, retention of donor and recipient samples, and arrangements for investigating unexplained illness, including after graft removal [34,35]. Programs need sustained microbiological capacity, traceability, and funding beyond the initial study. Surveillance of relevant contacts, donation restrictions, data access, and any public-health measures must be explained during consent and implemented under applicable law. Figure 2 summarizes the clinical pathway and its main risks.

## 6. Economics and Implementation Barriers

### 6.1. Costs and Cost-Effectiveness

Economic comparisons require a defined population, comparator, perspective, time horizon, and price basis, accounting for procedural, equipment, treatment, complication, and follow-up costs. Societal analyses also include travel, unpaid care, and lost work. Cost per quality-adjusted life-year (QALY), incremental cost per QALY gained, and budget impact address different questions. Table 2 summarizes the evidence and uncertainties. Table 2 summarizes the available economic evidence and its main uncertainties.

Economic evaluations generally favor human kidney transplantation over long-term dialysis in suitable patients, but their conclusions depend on recipient risk, graft survival, local costs, and the time horizon [36,40]. There is no universal break-even year. Comparisons between HD and PD are also sensitive to financing and consumable prices. An Indian lifetime model favored a PD-first policy from a societal perspective, whereas the direct-cost analysis depended strongly on PD consumable prices [38]. Such findings require reassessment before being applied in another healthcare system. The European EDITH report documented substantial cross-country variation in dialysis reimbursement tariffs using 2016 data; differences in the services covered by these tariffs limit direct comparisons of treatment costs [41].

The CONVINCE trial-based economic evaluation assessed HDF versus high-flux HD from a societal perspective. Its two-year analysis estimated incremental costs of €31,898–37,344 per QALY gained versus high-flux HD; lifetime extrapolation gave €27,068–36,751 per QALY, depending on staffing assumptions [37]. These estimates reflect alternative costing assumptions, rather than confidence intervals. The clinical comparison was randomized, whereas lifetime costs and outcomes were extrapolated using a model. Much of the additional expenditure arose from dialysis delivered during the additional years of survival. The results therefore require local costing and should not be interpreted as evidence of cost savings in every health system.

Economic evaluations of wearable and implantable devices should include all costs incurred throughout treatment, including device purchase and replacement, cartridges, electricity, maintenance, training, remote monitoring, and emergency care. Reduced facility use or travel may offset some expenditure, but these savings depend on device reliability and the support required. Reimbursement based on facility attendance or individual sessions may not adequately cover home training, technical support, or continuous treatment and could delay adoption.

For xenotransplantation, the available cost estimate by Vasudev and Cooper assumes eventual outcomes comparable to allotransplantation and identifies organ acquisition price as a major unknown [39]. It does not establish cost-effectiveness against dialysis. Future models should examine graft survival, immunosuppression, rejection, infection, lifelong surveillance, production scale, and organ price. They should also include graft failure, nephrectomy, return to dialysis, and subsequent transplantation. Favorable estimates based on prolonged graft survival remain conditional projections. Even lower costs per recipient could increase total expenditure if organ availability substantially expanded the number of patients treated.

### 6.2. Access, Workforce, and Financing

Financial and geographical barriers are already evident in established kidney care. In the 2023 ISN Global Kidney Health Atlas survey, only 74 of 166 countries reported public funding that made HD free at the point of delivery; nephrologist density differed approximately 80-fold between low- and high-income countries [42]. The national availability of dialysis services does not mean patients can reach or afford them. Out-of-pocket payments for sessions, drugs, transport, and temporary accommodation, together with lost income, can lead to catastrophic household spending or treatment interruption [43]. A rural patient may face repeated long-distance travel even when dialysis itself is reimbursed. Shortages of nurses, access surgeons, technicians, and transplant and laboratory staff further restrict delivery.

Home therapies require suitable housing, reliable utilities and supplies, training, and support. Xenotransplantation requires specialist surgery, immunology, pathology, microbiology, and prolonged surveillance. In settings with limited resources, these requirements may compete with essential services for staff and funding [40,42]. Public–private agreements should specify affordable access, transparent procurement, continuity of supplies and follow-up, and accountability for patient outcomes. Without such provisions, investment may favor profitable urban services while leaving poorer or rural populations underserved.

### 6.3. Consent, Cultural Concerns, and Prevention

Consent for xenotransplantation should explain its experimental status, uncertain graft durability, treatment burden, and the possibility of graft failure and return to dialysis. Patients with limited options may overestimate the likely benefit. Independent counseling, sufficient time for discussion, and continued access to usual care after declining participation can reduce undue influence.

FDA and US Public Health Service guidance recommend prolonged or lifelong surveillance and retention of donor and recipient samples. Consent should explain the implications for privacy, follow-up, and relevant close contacts, while distinguishing research participation from any applicable public-health obligations. Responsibility and funding for surveillance should be addressed beyond the initial study period [34]. Religious and cultural views about porcine organs vary within and between communities. Their acceptability should be discussed individually, with faith advisers if the patient wishes. Animal welfare, donor breeding practices, and public concerns also require transparent discussion.

Technological development should be matched by continued investment in the prevention and early treatment of CKD. Controlling diabetes and hypertension, treating primary kidney diseases, and providing timely kidney-protective care remain essential. Greater availability of dialysis or human and porcine transplantation cannot substitute for measures that prevent or delay kidney failure.

## 7. Limitations and Research Priorities

This narrative review does not provide an exhaustive search or a formal systematic assessment of study quality. Selective retrieval, publication bias, and uneven access to complete reports may affect its coverage. The evidence is particularly unstable for xenotransplantation and miniaturized devices, where small studies, selected patients, short follow-up, changing protocols, and sponsor reporting limit comparison. A trial protocol describes intended methods, and a cost model estimates outcomes under assumptions; neither should be treated as observed clinical effectiveness.

Kidney xenotransplantation studies need consistent definitions of patient survival, graft survival, death with a functioning graft, dialysis independence, rejection, serious infection, and return to dialysis. Harmonized surveillance and sample-retention arrangements should include responsibilities after graft failure and after a study ends. Dialysis-device studies should measure reliability during ordinary use, access events, patient and caregiver workload, and unplanned care. Both fields require patient-reported outcomes and economic models that test plausible graft or device lifetimes, complications, prices, and inequalities in access. Comparative research should assess whether benefits persist outside highly specialized centers.

## 8. Conclusions and Future Prospects

Despite these challenges, advances in dialysis and engineered porcine kidney transplantation are likely to be complementary rather than competitive. Dialysis will remain indispensable for acute kidney injury, for patients unsuited to any transplantation, and as a bridge to human or porcine grafts. Its evolution will likely emphasize incremental improvements in membrane biocompatibility, individualized dosing for small- and middle-molecule clearance, cardiovascular protection, closer control of fluid status and hemodynamic tolerance, prescriptions adapted to residual kidney function, greater use of home treatment and improved patient experience, alongside the gradual adoption of miniaturized and wearable technologies. Xenotransplantation, if ongoing trials confirm safety and medium-term efficacy, could be most impactful for older patients and those with prolonged dialysis vintage, who currently face high mortality and limited access to human organs.

Over the longer term, regenerative medicine, gene therapy, and improved prevention of kidney disease may reshape the incidence and demand for all forms of kidney replacement. For clinicians and investigators, the imperative is to integrate these innovations into coherent strategies that prioritize both equity and efficacy. Health-technology assessment must rigorously compare not only clinical outcomes but also full lifetime costs and opportunity costs, under realistic assumptions about uptake and survival. Public–private partnerships may be needed to ensure that a small number of firms do not monopolize the manufacturing of wearable dialysis devices and porcine organs, and that pricing allows their use beyond the highest-income health systems. Finally, an explicit global perspective is essential: without it, the future of dialysis, human kidney transplantation, and porcine kidney transplantation may become yet another example of cutting-edge medicine that deepens rather than bridges the divides among patients living with advanced kidney disease around the world.

## Figures and Tables

**Table 2:** Cost components, economic evidence, and key uncertainties across established and emerging kidney replacement therapies.

Modality	Setup Costs	Recurrent Costs	Economic Evidence	Horizon Perspective	Main Uncertainties
High-flux HD	Facilities, machines, water treatment, access	Staff, dialyzers and other supplies, quality assurance, vascular-access care, transport, and admissions	Comparator; costs vary by setting [36]	Long-term; payer or societal	Local prices, comorbidity, capacity
HvHDF	HDF machines, fluid quality, training	Same core cost categories as high-flux HD, with HDF-specific consumables and fluid-quality assurance requirements	€31,898–37,344/QALY (2 years); €27,068–36,751/QALY (lifetime), versus high-flux HD [37]	Two-year trial; lifetime model; societal	Survival extrapolation, added dialysis years
MCO HD	Standard HD equipment, fluid quality, training	Same core cost categories as high-flux HD, using MCO dialyzers, and fluid-quality assurance requirements	No cost–utility estimate included in this review	Lifetime evaluation needed	Dialyzer price, clinical benefit
Conventional PD	Catheter, training; cycler if used	Dialysate, delivery, support, complications	PD-first dominant in an Indian societal model [38]	Lifetime model; societal and direct-cost analyses	Consumable prices, technique survival, caregiver costs
Human kidney transplantation	Assessment, procurement, surgery	Immunosuppression, follow-up, complications, graft failure	Generally favored over dialysis in eligible patients [36]	Long-term; payer or societal	Graft survival, recipient risk, local costs
Wearable and implantable devices	Device, training; implantation if needed	Consumables, replacement, maintenance, monitoring	No long-term estimate in cited reports [15,17]	Patient’s treatment course; societal evaluation needed	Reliability, support needs, reimbursement
Porcine kidney transplantation	Donor engineering, breeding, procurement, surgery	Immunosuppression, surveillance, complications, graft failure	Preliminary cost estimate only [39]	Lifetime models needed; include public-health costs	Graft survival, organ price, production scale

HD, hemodialysis; HDF, hemodiafiltration; HvHDF, High-volume HDF; MCO, medium cut-off; PD, peritoneal dialysis; QALY, quality-adjusted life-year. HDF figures are incremental costs per QALY gained; ranges reflect staffing scenarios, not confidence intervals.

## Data Availability

No new data were created or analyzed in this review. Data sharing is not applicable.

## Abbreviations

The following abbreviations are used in this manuscript:

CMAH	CMP-N-acetylneuraminic acid hydroxylase (a porcine enzyme/gene edited to reduce xenoantigen expression).
CRISPR	Clustered Regularly Interspaced Short Palindromic Repeats (genome-editing system, used here as CRISPR–Cas9).
GGTA1	Alpha-1,3-galactosyltransferase 1 (porcine gene whose inactivation removes major xenoantigenic Gal epitopes).
B4GALNT2	Beta-1,4-N-acetylgalactosaminyltransferase 2 (porcine glycosyltransferase gene edited to reduce xenoantigenicity).
PERV	Porcine endogenous retroviruses.
HIC	High-income countries.
LMIC	Low- and middle-income countries.

## References

[1] National Library of Medicine (US) PubMed. https://pubmed.ncbi.nlm.nih.gov/.

[2] Ashby D., Borman N., Burton J., Corbett R., Davenport A., Farrington K., Flowers K., Fotheringham J., Andrea Fox R.N., Franklin G. (2019). Renal Association Clinical Practice Guideline on Haemodialysis. BMC Nephrol..

[3] Blankestijn P.J., Vernooij R.W.M., Hockham C., Strippoli G.F.M., Canaud B., Hegbrant J., Barth C., Covic A., Cromm K., Cucui A. (2023). Effect of Hemodiafiltration or Hemodialysis on Mortality in Kidney Failure. N. Engl. J. Med..

[4] Maduell F., Varas J., Ramos R., Martin-Malo A., Perez-Garcia R., Berdud I., Moreso F., Canaud B., Stuard S., Gauly A. (2017). Hemodiafiltration reduces all-cause and cardiovascular mortality in incident hemodialysis patients: A propensity-matched cohort study. Am. J. Nephrol..

[5] Peters S.A., Bots M.L., Canaud B., Davenport A., Grooteman M.P., Kircelli F., Locatelli F., Maduell F., Morena M., Nubé M.J. (2016). Haemodiafiltration and mortality in end-stage kidney disease patients: A pooled individual participant data analysis from four randomized controlled trials. Nephrol. Dial. Transplant..

[6] Vernooij R.W.M., Hockham C., Strippoli G., Green S., Hegbrant J., Davenport A., Barth C., Canaud B., Woodward M., Blankestijn P.J. (2024). Haemodiafiltration versus haemodialysis for kidney failure: An individual patient data meta-analysis of randomised controlled trials. Lancet.

[7] Strippoli G.F.M., Tripepi G., Canaud B., Stuard S., Maddux F.W., Usvyat L.A., Carioni P., Savoia M., Wong G., Zoccali C. (2026). Hemodiafiltration versus High-flux Hemodialysis and Risk of Mortality: A Multinational Target Trial Emulation. J. Am. Soc. Nephrol..

[8] Zhang Y., Winter A., Ferreras B.A., Carioni P., Arkossy O., Anger M., Kossmann R., Usvyat L.A., Stuard S., Maddux F.W. (2025). Real-world effectiveness of hemodialysis modalities: A retrospective cohort study. BMC Nephrol..

[9] Zhang Y., Winter A., Ficociello L.H., Ferrera B.A., Carioni P., Apel C., Arkossy O., Anger M., Kossmann R., Usvyat L.A. (2025). Real-World Hospitalization Outcomes with On-Line Hemodiafiltration Versus High-Flux Hemodialysis: A Retrospective, International Cohort Study. Clin. J. Am. Soc. Nephrol..

[10] Strogoff-de-Matos J.P., Canziani M.E.F., Barra A.B.L. (2025). Mortality on Hemodiafiltration Compared to High-Flux Hemodialysis: A Brazilian Cohort Study. Am. J. Kidney Dis..

[11] Canaud B., Bragg-Gresham J.L., Marshall M.R., Desmeules S., Gillespie B.W., Depner T., Klassen P., Port F.K. (2006). Mortality risk for patients receiving hemodiafiltration versus hemodialysis: European results from the DOPPS. Kidney Int..

[12] Zhang Y., Winter A., Ficociello L.H., Arya S., Stuard S., Usvyat L.A., Kalantar-Zadeh K. (2026). Hemodialysis Modality and Mortality Outcomes among Incident Dialysis Patients: An International Cohort Study Comparing High-Volume Hemodiafiltration and Hemodialysis. Clin. J. Am. Soc. Nephrol..

[13] de Sequera P., Pérez-García R., Vega A., Martínez-Vaquera S., Acosta J.G., Del Valle K.P., Fernández-Lucas M., García-Rubiales M.A., García-Herrera A.L., Coll E. MOTheR HDx study: A multicentre, open-label, prospective, randomized study to explore the morbidity and mortality in patients dialyzed with the Theranova HDx in comparison to online hemodiafiltration. Proceedings of the 63rd ERA Congress.

[14] Karkar A., Wilkie M. (2023). Peritoneal dialysis in the modern era. Perit. Dial. Int..

[15] Gura V., Rivara M.B., Bieber S., Munshi R., Smith N.C., Linke L., Kundzins J., Beizai M., Ezon C., Kessler L. (2016). A wearable artificial kidney for patients with end-stage renal disease. JCI Insight.

[16] de Vries J.C., van Gelder M.K., Bolhuis D.P., Simonis F., Verhaar M.C., Bajo Rubio M.A., Del Peso G., Selgas R., Donati G., Ligabue G. (2026). Rationale and design of the CORDIAL first-in-human clinical trial: A system for sorbent-assisted continuous flow peritoneal dialysis. Perit. Dial. Int..

[17] Rabb H., Lee K., Parikh C.R. (2022). Beyond kidney dialysis and transplantation: What’s on the horizon?. J. Clin. Investig..

[18] Kawai T., Williams W.W., Elias N., Fishman J.A., Crisalli K., Longchamp A., Rosales I.A., Duggan M., Kimura S., Morena L. (2025). Xenotransplantation of a Porcine Kidney for End-Stage Kidney Disease. N. Engl. J. Med..

[19] Riella L.V., Borges T.J., Rosales I.A., Avillach C.T., Palsson R., Hullekes F., Verhoeff R., Pomahac A., Chen J.Y., Santagata S. (2026). Porcine kidney xenotransplantation as a bridge to allotransplantation: A first-in-human study. Lancet.

[20] United Therapeutics First Transplant in EXPAND Clinical Trial of UKidney in Patients with End-Stage Renal Disease. Sponsor Announcement, 3 November 2025. Registry Identifier: NCT06878560. NCT06878560.

[21] eGenesis Clinical Update in Ongoing Expanded Access Study of EGEN-2784 in Patients with Kidney Failure. Sponsor Announcement, 3 September 2026. https://egenesisbio.com/news-media/press-releases/egenesis-announces-clinical-update-in-ongoing-expanded-access-study-of-egen-2784-in-patients-with-kidney-failure.

[22] Battaglia Y., Shroff R., Meijers B., Nistor I., Alfano G., Franssen C., Luyckx V., Liakopoulos V., Mantovani A., Baciga F. (2025). Haemodiafiltration versus high-flux haemodialysis—A Consensus Statement from the EuDial Working Group of the ERA. Nephrol. Dial. Transplant..

[23] Vernooij R.W., Strippoli G.F., Veltkamp D.M., Nistor I., Craig J.C., Saglimbene V.M., Covic A.C., Vecchio M. (2026). Haemodiafiltration, haemofiltration and haemodialysis for adults with kidney failure. Cochrane Database Syst. Rev..

[24] National Kidney Foundation (2015). KDOQI Clinical Practice Guideline for Hemodialysis Adequacy: 2015 update. Am. J. Kidney Dis..

[25] Murea M., Moossavi S., Garneata L., Kalantar-Zadeh K. (2020). Narrative Review of Incremental Hemodialysis. Kidney Int. Rep..

[26] Popovich R.P., Moncrief J.W., Nolph K.D., Ghods A.J., Twardowski Z.J., Pyle W.K. (1978). Continuous ambulatory peritoneal dialysis. Ann. Intern. Med..

[27] Roberts M. (1998). The regenerative dialysis (REDY) sorbent system. Nephrology.

[28] Fabiani T., Zarghamidehaghani M., Boi C., Dimartino S., Kentish S., De Angelis M.G. (2025). Sorbent-based dialysate regeneration for the wearable artificial kidney: Advancing material innovation via experimental and computational studies. Sep. Purif. Technol..

[29] O’Regan A., Thornton J., Clark E., Kant S. (2026). Xenotransplantation in Nephrology: A Narrative Review. J. Pers. Med..

[30] Yang L., Guell M., Niu D., George H., Lesha E., Grishin D., Aach J., Shrock E., Xu W., Poci J. (2015). Genome-wide inactivation of porcine endogenous retroviruses (PERVs). Science.

[31] Niu D., Wei H.J., Lin L., George H., Wang T., Lee I.H., Zhao H.Y., Wang Y., Kan Y., Shrock E. (2017). Inactivation of porcine endogenous retrovirus in pigs using CRISPR-Cas9. Science.

[32] Montgomery R.A., Stern J.M., Fathi F., Suek N., Kim J.I., Khalil K., Vermette B., Tatapudi V.S., Mattoo A., Skolnik E.Y. (2026). Physiology and immunology of a pig-to-human decedent kidney xenotransplant. Nature.

[33] Ribas G.T., Cunha A.F., Avila J.P., Giarraputo A., Morena L., Lima K., Gassen R.B., Chen J.Y., Lin J.R., Santagata S. (2026). Immune profiling in a living human recipient of a gene-edited pig kidney. Nat. Med..

[34] U.S. Food and Drug Administration Source Animal, Product, Preclinical, and Clinical Issues Concerning the Use of Xenotransplantation Products in Humans. Guidance for Industry, December 2016. https://www.fda.gov/media/102126/download.

[35] U.S. Public Health Service (2001). Guideline on Infectious Disease Issues in Xenotransplantation. MMWR Recomm. Rep..

[36] Wulandari W., Alfaqeeh M., Zakiyah N., Shafie A.A., Suwantika A.A. (2025). Cost-Utility Analyses of Hemodialysis, Peritoneal Dialysis, and Kidney Transplantation in Patients with End-Stage Kidney Disease: A Systematic Review. Clin. Outcomes Res..

[37] Schouten A.E.M., Fischer F., Blankestijn P.J., Vernooij R.W.M., Hockham C., Strippoli G.F.M., Canaud B., Hegbrant J., Barth C., Cromm K. (2025). A health economic evaluation of the multinational, randomized controlled CONVINCE trial: Cost-utility of high-dose online hemodiafiltration compared to high-flux hemodialysis. Kidney Int..

[38] Gupta D., Jyani G., Ramachandran R., Bahuguna P., Ameel M., Dahiya B.B., Kohli H.S., Prinja S., Jha V. (2022). Peritoneal dialysis-first initiative in India: A cost-effectiveness analysis. Clin. Kidney J..

[39] Vasudev K., Cooper D.K.C. (2025). How Much Will a Pig Organ Transplant Cost? A Preliminary Estimate of the Cost of Xenotransplantation Versus Allotransplantation in the USA. Xenotransplantation.

[40] Gadelkareem R.A., Abdelgawad A.M., Mohammed N., Zarzour M.A., Khalil M., Reda A., Hammouda H.M. (2024). Challenges to establishing and maintaining kidney transplantation programs in developing countries: What are the coping strategies?. World J. Methodol..

[41] Rahmel A., Lingemann M., Mihály S., Deme O., Bušić M., Dragović M., Jager K.J., Stel V.S., de Jong R., Cardillo M. (2020). EDITH—Final Public Report.

[42] Bello A.K., Okpechi I.G., Levin A., Ye F., Damster S., Arruebo S., Donner J.A., Caskey F.J., Cho Y., Davids M.R. (2024). An update on the global disparities in kidney disease burden and care across world countries and regions. Lancet Glob. Health.

[43] Yakubu A.O., Olusesi O.T., Lawal F.I., Abib O.M., Megbuwawon T.C., Olalude O.E., Bakare T., Almustapha H. (2025). Economic cost of end-stage renal disease in Africa: A systematic review. BMC Nephrol..

